# Sensor-Based Fault Diagnosis and Prognosis of Neurophysiological States: A Transformer Autoencoder Approach to EEG Monitoring

**DOI:** 10.3390/s26092913

**Published:** 2026-05-06

**Authors:** Jesús Jaime Moreno Escobar, Mauro Daniel Castillo Pérez, Erika Yolanda Aguilar del Villar, Hugo Quintana Espinosa

**Affiliations:** 1 Centro de Investigación en Computación, Instituto Politécnico Nacional, Ciudad de México 07738, Mexico; jmorenoe@ipn.mx; 2 Escuela Superior de Ingeniería Mecánica y Eléctrica, Unidad Zacatenco, Instituto Politécnico Nacional, Ciudad de México 07340, Mexico; eaguilard2000@alumno.ipn.mx (E.Y.A.d.V.); hquintana@ipn.mx (H.Q.E.); 3 Escuela Superior de Cómputo, Instituto Politécnico Nacional, Ciudad de México 07320, Mexico

**Keywords:** unsupervised representation learning, transformer autoencoders, latent space analysis, electroencephalography, dolphin-assisted therapy

## Abstract

This study presents a sensor-based condition monitoring framework for the diagnosis and prognosis of neurophysiological states using electroencephalographic (EEG) signals. Leveraging a comparative deep learning architecture, we evaluate a baseline Variational Autoencoder against a Transformer-based Autoencoder to model latent representations of EEG dynamics across three therapeutic phases: pre-intervention, during intervention, and post-intervention. The proposed methodology aligns with sensor-based fault diagnosis principles by treating deviations from stable neurophysiological states as diagnostic indicators and temporal phase transitions as markers of therapeutic stage progression. Using a dataset of 94 EEG sessions from six subjects with diverse neurological conditions, we demonstrate that the Transformer Autoencoder, through its self-attention mechanism, captures cross-band spectral relationships more effectively than the VAE, resulting in denser within-phase clusters and improved separation between therapeutic stages. Quantitative evaluation reveals small but statistically significant effects between pre- and during-intervention phases ($\eta_{\mathrm{partial}}^{2}=0.0388$) and pre- and post-intervention phases ($\eta_{\mathrm{partial}}^{2}=0.0470$), predominantly driven by delta, theta, beta, and gamma rhythms. These findings illustrate how sensor-based latent state monitoring can provide interpretable, data-driven insights for condition assessment and phase transition assessment between sessions in complex dynamic systems, with potential applicability beyond clinical domains to industrial condition monitoring and fault diagnosis tasks. The framework confirms that it offers qualitative indicators, rather than predictive clinical outputs.

## 1. Introduction

This study has implications for an emerging social and clinical challenge: enhancing the monitoring and follow-up of therapeutic processes in populations receiving wellness-oriented therapeutic interventions, for which assessment is often subjective or constrained by cost and availability. In this regard, dolphin-assisted therapy has received attention as an experimental neurorehabilitative therapy and is associated with positive outcomes; however, its neurophysiological response is still poorly defined across various neurological conditions. Unfortunately, there are no objective and interpretable indicators to assess therapy-induced changes, which can hinder clinical decision-making and inhibit sustained therapeutic continuity. Societally, this study aspires to contribute to quantitative methodologies designed to quantify processes to trace a therapeutic trajectory via the intertwining of subjective experience and objective neurophysiological measurements. Electroencephalography (EEG) is a non-invasive technique for recording brain activity at a high temporal resolution and can provide a valuable insight into cognitive and neurophysiological states. EEG analysis is typically limited to short-term measures, and recent research has shown its promise for long-term measurements of subtle neurophysiological alterations. In this study, EEG signals are monitored in the long-term (before therapy, during treatment, and after therapy) to characterize differences in therapeutic changes from other treatments and gain an additional point of view beyond just present moment measurement. Here, a fault is a deviation from the neurophysiological set of patterns usually present for stable cognition or therapy. Instead of a mechanical failure, the faults refer to abnormal or variable brain activity that could indicate changes in patient response during therapy. Thus, fault diagnosis could be analyzed as the detection of deviations in EEG representations, and prognosis as the predictive interpretation of temporal trends on those deviations. Technically speaking, the difficult part is learning stable and expressive latent representations from single channel EEG signals, which are noisy, variable, and subject sensitive. To solve this issue, we introduce an autoencoder-driven representation-learning framework that represents EEG dynamics at various therapy levels. Namely, we compare a reference variational autoencoder (VAE) with a transformer-based autoencoder with respect to their ability to preserve the structure of brain rhythms and the ability of both to perform the temporal analysis. Although recent developments in transformer-based tools have significantly improved the performance of EEG models, most current approaches are either baseed on supervised classification or short-term task signaling interpretation tasks. Alternatively, to model long-term neurophysiological dynamics, we propose an unsupervised representation learning framework. Uncovering latent patterns that arise as therapy proceeds therefore provides a novel perspective for EEG-based monitoring and anomaly interpretation in therapeutic applications. Autoencoders are unsupervised neural network models that reconstruct key information from this input data through a compressed latent space. Autoencoders typically involve two main parts: an encoder that scales dimensions and extracts necessary features, and a decoder that reconstructs the input based on the latent representation [1]. These models are particularly suitable for capturing complex and nonlinear structures in EEG signals. To further aid interpretation of the obtained representations, dimensionality reduction methodologies, including UMAP (Uniform Manifold Approximation and Projection), have been used. UMAP is a nonlinear technique that allows high-dimensional data visualization in lower-dimensional space and preserves local and global structures. By providing a graph-based representation of neighborhood relations, UMAP enables exploration of areas not always detected in EEG networks, clustering, and identification of complex patterns in data [2].

In past years, electroencephalographic (EEG) signals have been extensively analyzed significant using deep learning methods. In contrast to mainstream techniques, such as the Fast Fourier Transform (FFT) or autoregressive methods, which are based on manual feature extraction and assume stationarity of the signal, DL-based methods are able to implicitly model nonlinear, non-stationary, and highly noisy EEG signals. In this line of research, recent reviews [3,4] have shown that neural networks, such as CNN, RNN, and Transformer architectures, have greatly improved accuracy in clinical tasks, including seizure detection, sleep data, and neurological diagnosis. Autoencoders (AE) have emerged as a fundamental class of unsupervised learning method in these approaches to extract the latent representations. Specifically, variational autoencoders (VAE) have demonstrated a high capacity for capturing complex distributions of information across a wider range of features in EEG signals, where they can effectively extract relevant features even from a limited amount of labeled data [5]. Similarly, ref. [6] has further expanded upon this work, including constraints such as sparsity and interpretability, and hybrid architectures that further facilitate the performance of certain clinical tasks. Moreover, based on different studies of autoencoder ensembles [7] and recent methods, combining several models allows the capture of various aspects of normality, improving the robustness of anomaly detection in biomedical data. However, EEG remains a strongly temporal problem. A notable limitation of many autoencoder-based approaches is that static representations dominate their capabilities for modeling long- and short-term dynamic dependencies. Multiple works have proposed models with hierarchical temporal convolutions and multi-resolution methods to minimize these issues and better simulate highly intricate neural dynamics [8]. This evolution has led to the development of neural-network combinations that enhance models that capture temporal and structural information in parallel. Transformer-based architectures are promising candidates for such an effort, as they can model long-range dependencies with self-attention mechanisms. In the literature [9] it has been demonstrated that these models are also utilized for applications such as emotion classification, seizure detection, and motor imagery analysis. Similarly, works such as [10,11] demonstrate that Transformers can represent spatial and temporal information as spatio-temporal features, which is advantageous over existing approaches. Hybrid methods combining autoencoders and Transformers with representations and temporal modeling have been proposed more recently. An example is the proposed CwA-T model [12], which maintains separation between EEG signals using a channel-wise convolutional autoencoder, which can then be augmented by a Transformer to model temporal dependencies, thereby finding a good balance between accuracy and interpretability. Likewise, the CAE-T model [13] combines a convolutional autoencoder with a Transformer and an optimization mechanism derived from SVDD, and has substantially improved performance in complex industrial systems. For example, in the area of anomaly detection in multivariate time series, VTT [14] has proposed two attention mechanisms that capture both temporal dependencies and correlations between variables, and the enhanced pre-trained Transformer (TEP) [15] has been proposed and is coupled with convolutional modules for better contextualization in the local context space. These developments have shown that the integration of local and global data is necessary to enhance anomaly event detection. However, the limited amount of labeled data is still a challenge in EEG-based models. To counter this issue, a number of approaches, including data augmentation [16], self-supervised learning, and the employment of shared latent representations [17], have been proposed, each of which aims to increase the generalization capability of an effective model on a small volume of data. Last but not least is model interpretation. In crucial instances such as clinical diagnosis or industrial systems, there is a requirement not only to detect anomalies but also understand their underlying causes. The gray-box fault diagnosis framework, which is based on Kolmogorov–Arnold networks and decision trees [18] has been demonstrated in some aspects of this work that it is possible to have both predictive accuracy and interpretability, giving clear rules for decision-making. Similarly, recent efforts in computational neuroscience have presented closed-loop and adaptive schemes containing EEG acquisition, brain state modeling, and intervention selection [19]. Although impressive approaches with autoencoders, Transformers, and hybrid models have been developed, significant limitations still exist. Specifically, a great number of approaches are unable to effectively combine representation learning, temporal modeling, and interpretability, particularly in the case of small amounts of data and highly noisy signals such as EEG. This calls for the design of architectures that combine such capabilities holistically, and this is the motivation for the approach proposed here.

This paper is organized as follows. Section 2 presents the theoretical foundations of EEG analysis, describing its non-stationary and noisy nature, as well as its representation and frequency characteristics. It introduces fault diagnosis and anomaly detection using reconstruction methods such as autoencoders and VAEs. In addition, this section describes the dataset, including its distribution and the exploratory analysis performed, the proposed methodology, detailing the architecture based on autoencoders and Transformers, as well as the tools used for its implementation. Then, Section 3 describes the experimental setup, the conducted tests, and analyzes the results obtained from the combination of autoencoders and Transformers. In Section 4, a comprehensive analysis of multivariate EEG comparisons is presented along with the limitations of this proposal. Finally, Section 5 summarizes the main findings of the study and outlines directions for future research.

## 2. Materials and Methods

### 2.1. Fault Diagnosis in EEG-Based Systems

**Figure 1:**
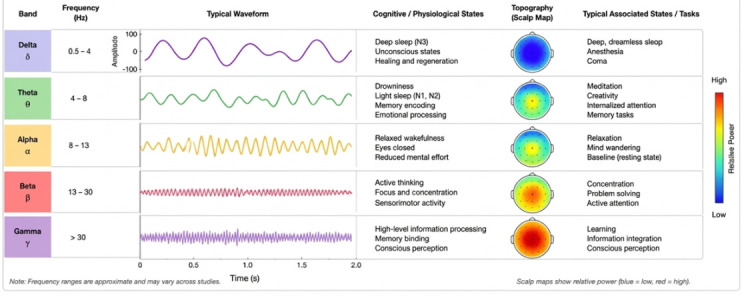
Representation of standard EEG frequency bands (delta, theta, alpha, beta, and gamma) and their associated cognitive states.

Electroencephalographic (EEG) signals are electrical recordings of brain activity acquired through electrodes placed on the scalp. These signals reflect the collective behavior of neuronal populations over time and are characterized by their non-stationary nature, high complexity, and low signal-to-noise ratio. One of the main advantages of EEG signals is their high temporal resolution, which allows the capture of rapid neural dynamics on the order of milliseconds. However, EEG signals are highly susceptible to various sources of noise and artifacts, including ocular movements, muscle activity, and environmental interference, which complicates their analysis. EEG signals are also commonly analyzed in the frequency domain, where they are decomposed into characteristic bands such as delta (0.5–4 Hz), theta (4–8 Hz), alpha (8–13 Hz), beta (13–30 Hz), and gamma (>30 Hz). These frequency bands are associated with different cognitive and physiological states, making spectral analysis a key component in EEG-based studies, as shown in Figure 1.

**Figure 2:**
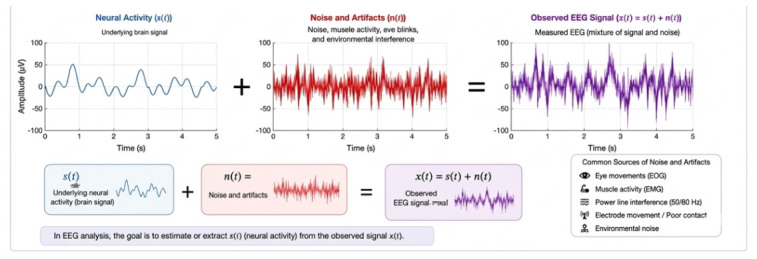
Mathematical model of EEG signals showing the decomposition into neural activity s(t) and noise n(t), forming the observed signal x(t). This representation highlights the complexity and noise sensitivity of EEG data, (Author’s own elaboration).

Mathematically, an EEG signal can be modeled as:

(1) x(t)=s(t)+n(t)

where s(t) represents the underlying neural activity and n(t) denotes noise and artifacts. Due to these characteristics, advanced signal processing and machine learning techniques are required to extract meaningful patterns from EEG data, as shown in Figure 2.

On the one hand, Fault diagnosis refers to the process of detecting, identifying, and classifying abnormal conditions in a system. In the context of EEG analysis, it can be interpreted as identifying anomalous neurophysiological states or deviations from normal brain activity.

Formally, fault diagnosis can be defined as a mapping:

(2) D:X→Y

where *X* represents the observed data (e.g., EEG signals) and *Y* corresponds to the system states (e.g., normal or abnormal conditions).

Different approaches have been proposed for fault diagnosis:

- Supervised methods: require labeled data to train classification models.
- Unsupervised methods: identify anomalies without labeled data.
- Feature-based methods: rely on handcrafted features such as statistical measures or spectral energy.
- Deep learning approaches: automatically learn hierarchical representations from raw data.

Anomaly detection aims to identify patterns that significantly deviate from normal behavior. It plays a central role in fault diagnosis, particularly when labeled data are scarce. A common strategy is to model the distribution of normal data and detect anomalies as deviations:

(3) xisconsideredanomalousifp(x)<ϵ

where p(x) is the probability density function and ϵ is a predefined threshold.

Reconstruction-based methods, such as autoencoders, have gained significant attention. These models learn to reconstruct input data, and anomalies are detected through reconstruction error:

(4) $$E=∥x-\hat{x}∥$$

where *x* is the original input and $\hat{x}$ is its reconstruction.

Variational Autoencoders (VAEs) extend this idea by modeling latent representations probabilistically. Unlike standard autoencoders, VAEs impose a structured distribution over the latent space by approximating the posterior distribution of latent variables. This is achieved by optimizing the evidence lower bound (ELBO):

(5) $$L=E_{q(z|x)}\left[\log p\left(x|z\right)\right]-D_{KL}\left(q\left(z|x\right)∥p\left(z\right)\right)$$

where $D_{KL}$ denotes the Kullback–Leibler divergence. This formulation encourages the latent space to follow a predefined distribution, typically Gaussian, improving generalization and robustness to noise.

This property is particularly relevant for EEG signals, which exhibit high variability, uncertainty, and non-stationary behavior. In EEG analysis, high reconstruction errors may indicate abnormal brain activity or transitions between cognitive states.

On the other hand, representation learning focuses on automatically extracting meaningful features from raw data, eliminating the need for manual feature engineering.

A model learns a transformation:

(6) z=f(x)

where *z* is a latent representation capturing essential information about the system.

These representations provide several advantages:

- Dimensionality reduction
- Automatic feature extraction
- Improved modeling of complex temporal dependencies

In EEG-based systems, latent representations enable capturing hidden neurophysiological patterns that are not directly observable in the original signal space.

On the other hand, Transformer architectures have emerged as powerful models for capturing long-range dependencies in sequential data. Unlike recurrent models, Transformers rely on attention mechanisms to model relationships between different time steps.

Given the temporal nature of EEG signals, Transformers are particularly suitable for capturing complex dynamics and dependencies across time, improving the quality of learned representations.

In this work, a Transformer-based autoencoder is employed to enhance the modeling of EEG temporal structures within the latent space.

The concepts described above are integrated into a unified framework:

- Anomaly detection supports fault diagnosis by identifying deviations from normal EEG patterns.
- Latent representations encode different system states, including normal and abnormal conditions.
- Reconstruction error provides a quantitative measure for detecting anomalies.
- Transformer-based architectures improve the modeling of temporal dependencies in EEG signals.

Despite advances in EEG analysis, challenges remain due to noise, high dimensionality, and complex temporal dependencies. Traditional approaches often fail to simultaneously capture probabilistic representations and long-range temporal patterns.

Therefore, this work proposes a unified framework that combines variational inference and Transformer-based architectures to address these limitations in a principled manner. This integration forms the theoretical foundation of the proposed approach, in which representation learning and reconstruction-based anomaly detection are combined to analyze neurophysiological dynamics.

This theoretical framework provides the basis for the exploratory analysis presented in the next section, in which the statistical, temporal, and spectral properties of EEG signals are examined prior to model construction.

### 2.2. Database Description

**Figure 3:**
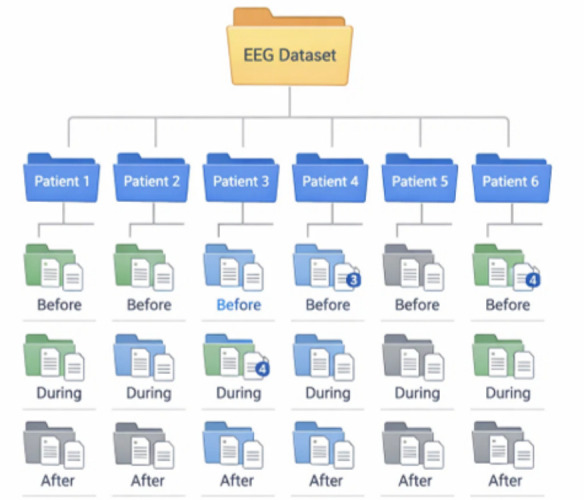
EEG Dataset Structure.

The research uses a set of 94 EEG data files, systematically organized by participant, experimental moment, and year of acquisition. The data correspond to six subjects identified as Patient 1, Patient 2, Patient 3, Patient 4, Patient 5, and Patient 6, which allows a comparative analysis both inter-subject and longitudinally. This database is shown in Figure 3. In terms of distribution by patient, the participants have similar numbers of files, although with slight variations. Patient 3 is the subject with the highest number of records, with 19 files, followed by Patient 2 with 17 files, Patient 6 with 16 files, and finally Patient 4 with a total of 15 files. Meanwhile, Patient 5 has 14 files and Patient 1 has 13 files; these last two patients have a slightly lower number of files. These differences are not considered extreme and allow for a relatively balanced experimental design among participants. From the perspective of the moments at which the EEG signal was acquired, the records are grouped into three categories: Before, During, and After. The Before moment has the highest number of files, with 36, while During and After each have 29 files. The .zip file containing this described database can be obtained from the following link: EEG_DAT_Patients.zip (https://drive.google.com/file/d/1WLPe6PFv67Twize7NcNNFMeKMaAOyLse/, accessed on 2 May 2026). The dataset consists of anonymized demographic data from six individuals recruited through the Delfiniti study. Subjects comprised one female and five males, aged 10 to 29 years, diagnosed with cerebral palsy, Down syndrome and autism spectrum disorder (including one case of grade II autism). During the study time frame, all participants were accompanied by their mothers. Socioeconomic position was inferred from the area of residence (Zihuatanejo, Guerrero, Mexico): low for eight participants and lower middle for one participant. All participants underwent eight therapy sessions. It is important to acknowledge as a study limitation that detailed medical histories and medication use were not available because of anonymization protocols. An IRB (Institutional Review Board) reviewed the study and deemed it ethically appropriate. The approval for this work was received from the Ethics Committee of the National Polytechnic Institute of Mexico (Instituto Politécnico Nacional, IPN) with protocol reference D/1477/2020.

This distribution allows for greater emphasis on baseline measurement while maintaining an adequate balance between the active and subsequent phases of the experiment. In addition, each file corresponds to acquisitions collected across three years: 2022, 2023, and 2024, providing a relevant temporal dimension within the data. Early records from 2022 for some participants were expanded in 2023, followed by a denser and more systematic block in 2024, specifically across the three moments at which the EEG signal was obtained; this structure allows for the evaluation of stability, variability, and possible long-term effects in the EEG signals. In conclusion, for the preparation of this research, a series of robust, well-labeled, and methodologically consistent data were used, which makes the data suitable for comparative analysis among patients and among the various moments at which the EEG signal was acquired (Before, During, and After), thus allowing the measurement of changes associated with therapy and the assessment of cognitive state through multivariate analysis.

### 2.3. Cross-Session Evolution of EEG Bands

**Figure 4:**
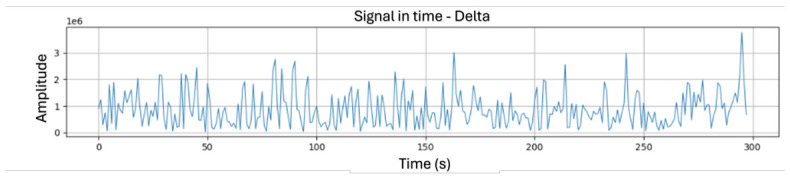
Patient 3 Delta frequency signal over time.

By obtaining the patients’ EEG frequencies, an exploratory analysis of the cross-session evolution of EEG bands was performed. These bands show consistent patterns throughout the recordings; an example of these bands can be seen in Figure 4. Every recording was saved in a text file and then zipped. Delta (δ, 0.5–2.75 Hz), theta (θ, 3.5–6.75 Hz), low-alpha ($\alpha_{↓}$, 7.5–9.25 Hz), high-alpha ($\alpha^{↑}$, 10–11.75 Hz), low-beta ($\beta_{↓}$, 13–16.75 Hz), high-beta ($\beta^{↑}$, 18–29.75 Hz), low-gamma ($\gamma_{↓}$, 31–39.75 Hz), high-gamma ($\gamma^{↑}$, 41–49.75 Hz), along with attention and meditation stimuli were analyzed. During the exploration of these bands, it was observed that individuals with low frequencies (δ and θ) had stronger amplitude and greater temporal variability, manifested as isolated bursts or peak transience. This reflects a slow and dominant dynamic associated with both global regulatory processes and internal states. Unlike the low-frequency bands, e.g., $\gamma_{↓}$ and $\gamma^{↑}$, or medium-frequency bands like $\alpha_{↓}$ and $\alpha^{↑}$; or $\beta_{↓}$ and $\beta^{↑}$ which capture lower peaks in amplitude, there is a less regular signal in the high-frequency regime characterized by transient enveloped signals within which there are bursts that signify sensory-cognitive integration. All of these generate attention and meditation metrics, which present a smoother and more contiguous temporal evolution compared to other EEG bands that have no extreme peaks or sudden oscillations. These mechanisms provide a global perspective on the functional organization of neuronal activity, since the temporal characterization of different EEG bands can also discriminate between slow regulatory phenomena and fast brain-processing and integrating mechanisms. The identified temporal patterns indicated the plausible presence of structural spectral determinants that warrant a correlation analysis between the distinct frequency bands and cognitive metrics.

### 2.4. Spectral Structure and Relationships Between Variables

**Figure 5:**
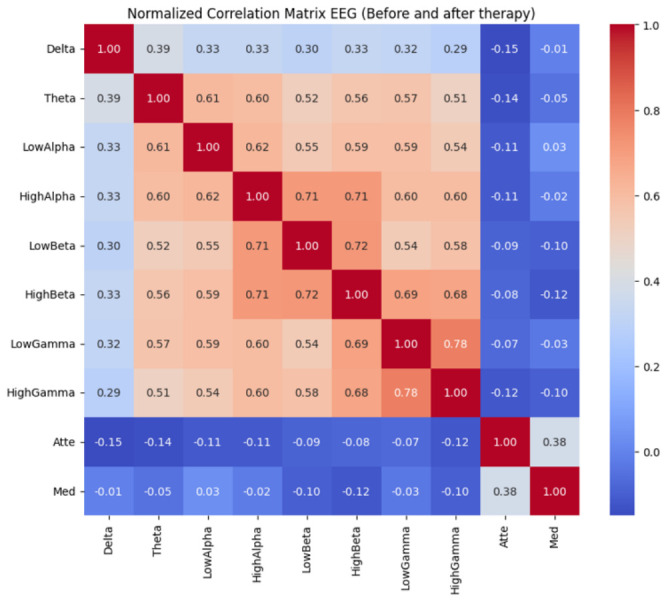
Correlation matrix.

To observe the effect of these frequency bands, the normalized EEG correlation matrix shown in Figure 5 was examined by considering the combination of the recording time with the comparison time before and after therapy. This matrix clearly shows a distinct hierarchical structure with respect to the various frequency bands. This indicates spectral co-modulation and coordinated cortical activation, with moderate to high positive correlations among α, β and γ. Importantly, the high correlations between frequencies in adjacent subbands, e.g., $\beta_{↓}$–$\beta^{↑}$ and $\gamma_{↓}$–$\gamma^{↑}$, indicate that these frequencies do not operate independently but are part of a coupled neuronal process. The slow bands, especially δ and θ, correlate more moderately with the rest of the spectrum, indicating a somewhat differentiated pattern of functional behavior. These experiments explore the relationship and suggest that low-frequency EEG is associated with global regulatory processes and internal states, in contrast to higher-frequency bands, which tend to be related to engaged cognitive processing. Attention- and meditation-related frequencies are weakly negatively correlated with other EEG bands, suggesting that these frequencies were not linearly dependent on the power of other bands. In conclusion, the correlation matrix with frequencies measured before and after therapy shows that the intervention does not alter the overall spectral organization of the EEG but rather modulates the intensity or dynamics of the bands. With these general spectral characteristics obtained, the dominant patterns can be analyzed using dimensionality reduction techniques such as PCA.

### 2.5. Principal Component Analysis

To identify the dominant patterns of variability between frequencies and to reduce the dimensionality of the EEG dataset, principal component analysis (PCA) was applied using the EEG frequency bands and the cognitive metrics of attention and meditation, integrating the recordings obtained before and after therapy.

**Figure 6:**
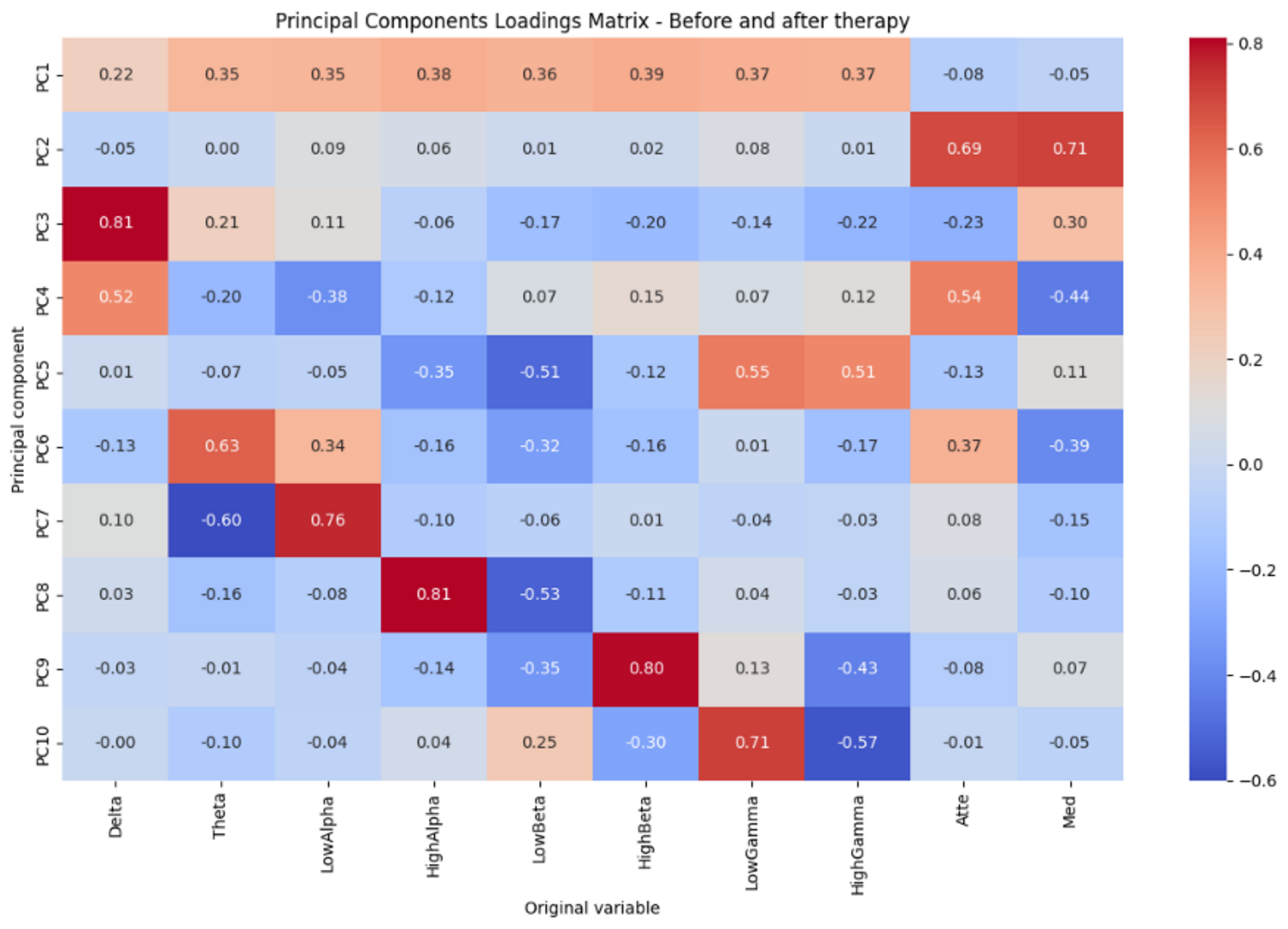
Principal component loading matrix showing the relative contribution of EEG bands and attention and meditation metrics.

Figure 6 shows the loading matrix of the principal components. Each component represents a linear combination of the original variables and allows for the interpretation of the relative contribution of each frequency band and cognitive metric to the observed patterns of variability. The first principal component (PC1) exhibits moderate and homogeneous positive loadings in the α, β, and γ bands, while the contributions of attention and meditation are low and negative. This pattern suggests that PC1 describes an overall spectral activation dominated by mid and high frequencies, reflecting a general level of cortical activity independent of the derived cognitive indices. In contrast, the second principal component, PC2, is characterized by high and positive loadings in the attention and meditation metrics, with minimal participation from the other EEG bands; this indicates that PC2 captures a global cognitive state, distinct from direct spectral power. This reinforces the idea that these metrics represent integrative processes and are not dependent on a single frequency band. The PC3 and PC4 components show a strong influence in the δ band, accompanied by variable contributions from α and attention. These components can be interpreted as patterns associated with slow fluctuations in brain activity, possibly related to internal states, global regulation, or transitions between different levels of cortical activation. On the other hand, components PC5 through PC7 show a combination of opposing loadings between the β and γ bands, indicating differential relationships between mid- and high-frequency bands. These patterns suggest mechanisms of spectral balance and functional coupling, potentially linked to more complex dynamics of cognitive processing. Finally, the higher-order components from PC8 to PC10 are dominated by specific bands, such as $\alpha^{↑}$, $\beta^{↑}$, or γ, capturing more local variations of the EEG spectrum that, although they explain a smaller proportion of the total variance, may contain relevant information at the individual level.

**Figure 7:**
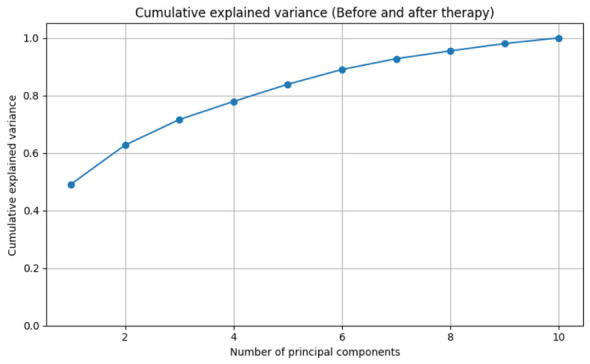
Cumulative variance explained by the principal components, indicating that a small number of components capture most of the variability in the data.

On the other hand, Figure 7 presents the cumulative variance explained by the principal components. It can be seen that the first component explains a high proportion of the total variance, close to 50%, indicating the presence of a dominant pattern in the EEG data. The addition of the second and third components increases the cumulative explained variance to approximately 60–70%, suggesting that much of the relevant information can be represented in a low-dimensional subspace. From the fourth and fifth components onward, the increase in explained variance becomes more gradual, reaching around 80% with four components and exceeding 85% with five. Finally, with six to seven components, the cumulative explained variance exceeds 90%, while the remaining components make marginal contributions.

Based on these results, the analysis can be extended to more complex latent representation models, allowing the exploration of deep architectures based on autoencoders and Transformers, which can capture underlying EEG structures that are not evident using linear techniques such as UMAP.

### 2.6. Baseline Variational Autoencoder

To begin with, we use a straightforward probabilistic model to specify both a hidden representation and a reconstruction objective before considering more intricate models. Instead of an ad hoc replication experiment, this baseline is a well-defined matched filter reference that we may use to measure how much the structure of the EEG rhythm features can be retrieved with the smallest generative model. In the process, it represents a fair and interpretable benchmark for comparing its latent-space quality, stability, and reconstruction error with those obtained by more sophisticated models [5]. We then extend this approach to Transformer-based autoencoders. The self-attention mechanism provides a more flexible manner of modeling global dependencies and interactions between features, which is highly applicable to EEG rhythms given that they can display non-local correlations and strong subject-dependent variability. Transformer architectures are not so limited and can potentially learn more complex or richer encodings for the latent representations, since they scale better to non-homogeneous patterns, support end-to-end training, and perform well with modern regularization techniques, compared to classical autoencoders or classical VAEs [20].

We adopted a baseline variational autoencoder to compress EEG rhythm features into latent representation. Each recording was saved in a text file and then zipped. From these files, we extracted the brain rhythms, such as δ, θ, $\alpha_{↓}$, $\alpha^{↑}$, $\beta_{↓}$, $\beta^{↑}$, $\gamma_{↓}$, and $\gamma^{↑}$, together with attention and meditation stimuli, converted them to numeric values, and discarded invalid entries. We then summarized each session by taking the average over the available observations. This led to a characteristic matrix $X\in R^{N\times 10}$, with *N* representing the sessions.

All features were standardized using z-score normalization, as follows:

(7) $$X_{n}=\frac{X-\mu }{\sigma },$$

where μ and σ are the mean and standard deviation per-feature computed on the data set.

**Figure 8:**
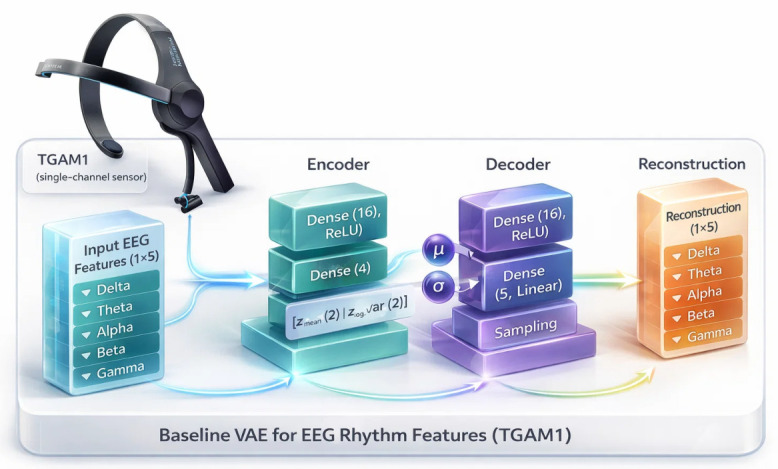
Baseline VAE for EEG rhythm features from the TGAM1 single-channel sensor. The encoder maps the 5-band input (Delta, Theta, Alpha, Beta, Gamma) through Dense (16, ReLU) to a 2D latent distribution (μ,σ) and is sampled to reconstruct the 5-band output through a symmetric decoder.

From Figure 8, the proposed VAE has an encoder and a decoder of 16 hidden units each. The encoder maps the input to the parameters of a diagonal Gaussian posterior:

(8) $$q_{\phi }\left(z|x\right)=N\;\left(z;\;\mu_{\phi }\left(x\right),\mathrm{diag}\left(\sigma_{\phi }^{2}\left(x\right)\right)\right),$$

where $\mu_{\phi }\left(x\right)$ and $\log\sigma_{\phi }^{2}\left(x\right)$ are the output of a Dense(4) layer and are then divided as two vectors of dimension 2. The latent samples are obtained via the reparameterization trick described as:

(9) $$z=\mu_{\phi }\left(x\right)+\sigma_{\phi }\left(x\right)⊙\epsilon ,\;\epsilon ∼N\left(0,I\right).$$

The decoder reconstructs the input from z with a symmetric architecture giving $\hat{x}=f_{\theta }\left(z\right)$ with a linear output layer. We train the model with the standard VAE objective:

(10) $$L\left(\theta ,\phi ;x\right)=\underset{\mathrm{reconstruction}\;\left(\mathrm{MSE}\right)}{\underset{︸}{∥x-\hat{x}{∥}_{2}^{2}}}+\underset{\mathrm{regularization}}{\underset{︸}{D_{\mathrm{KL}}\;\left(q_{\phi }\left(z|x\right)\;∥\;N\left(0,I\right)\right)}},$$

where the KL term of the Diagonal Gaussian takes the closed form:

(11) $$D_{\mathrm{KL}}=-\frac{1}{2}\sum_{j=1}^{2}\left(1+\log\sigma_{j}^{2}-\mu_{j}^{2}-\sigma_{j}^{2}\right).$$

The network was trained for 200 epochs using Adam optimization, with a batch size of 8. This size was used to balance gradient stability and generalization on a small EEG dataset with low GPU memory requirements, while remaining compatible with frequent parameter updates useful for the VAE to converge without overfitting. After training, the mean vector $\mu_{\phi }\left(x\right)\in R^{2}$ of the encoder was used as the two-dimensional latent representation for each session.

### 2.7. Transition to Transformer Autoencoder

Each session was summarized from zipped text files containing eight rhythm bands—delta (δ, 0.5–2.75 Hz), theta (θ, 3.5–6.75 Hz), low-alpha ($\alpha_{↓}$, 7.5–9.25 Hz), high-alpha ($\alpha^{↑}$, 10–11.75 Hz), low-beta ($\beta_{↓}$, 13–16.75 Hz), high-beta ($\beta^{↑}$, 18–29.75 Hz), low-gamma ($\gamma_{↓}$, 31–39.75 Hz), high-gamma ($\gamma^{↑}$, 41–49.75 Hz)—together with two stimulus-related features (attention and meditation).

The base VAE presented a compressed and interpretable latent space for the EEG rhythm features, while also revealing pragmatic limitations in covering the entire structure of the data. Although the VAE performed well as a probabilistic baseline, its MLP-based encoder–decoder structure was naturally constrained in capturing intricate cross-band interactions and subject-specific variations. Therefore, the reconstructions may have been too smooth, and the learned latent space might discard higher-order statistical dependencies of EEG signals. To mitigate these constraints, we moved to a Transformer-based autoencoder using self-attention mechanism for encoding global dependencies between rhythm bands and stimulus features without assuming locality or fixed interactions. This architecture can capture adaptive feature interactions and is well-suited to the diverse set of non-stationary, local, and non-local patterns often present in EEG recordings. When shallow VAE architectures such as traditional feedforward networks are used, Transformer autoencoders will result in richer latent features and better stability, while still supporting probabilistic latent representations for downstream tasks.

### 2.8. Transformer Autoencoder Design

The final autoencoder replaces the MLP bottleneck with a lightweight transformer encoder to better model cross-band interactions among the EEG rhythms and stimuli. Each session is represented by a standardized input vector $x\in R^{d}$, where d=10 corresponds to eight rhythm bands (delta, theta, low-/high-alpha, low-/high-beta, low-/high-gamma) plus attention and meditation. The encoder first projects the input to a 64-dimensional embedding. Each session was summarized from zipped text files containing eight rhythm bands—delta (δ, 0.5–2.75 Hz), Theta (θ, 3.5–6.75 Hz), low-slpha ($\alpha_{↓}$, 7.5–9.25 Hz), high-alpha ($\alpha^{↑}$, 10–11.75 Hz), low-beta ($\beta_{↓}$, 13–16.75 Hz), high-beta ($\beta^{↑}$, 18–29.75 Hz), low-gamma ($\gamma_{↓}$, 31–39.75 Hz), high-gamma ($\gamma^{↑}$, 41–49.75 Hz)—together with two stimulus-related features (attention and meditation).

In this way, this autoencoder replaces the MLP bottleneck with a compact transformer encoder to better exploit cross-band interactions between EEG rhythms and stimulus-related features. A session is described by a feature vector $x\in R^{d}$, where d=10 represents the 8 rhythm sub-bands: δ, θ, $\alpha_{↓}$, $\alpha^{↑}$, $\beta_{↓}$, $\beta^{↑}$, $\gamma_{↓}$ and $\gamma^{↑}$, in addition to attention and meditation. First, the encoder maps this input into a 64-dimensional space.

(12) $$h=\phi (W_{1}x+b_{1}),\;\phi =\mathrm{ReLU},$$

and applies multi-head self-attention then to capture global dependence across features:

(13) $$\mathrm{MHA}\left(H\right)=\mathrm{Concat}\left({\mathrm{head}}_{1},\ldots ,{\mathrm{head}}_{m}\right)W^{O},$$

where each head is defined as:

(14) $$\begin{array}{c} {\mathrm{head}}_{i}=\mathrm{softmax}\;\left(\frac{Q_{i}K_{i}^{⊤}}{\sqrt{d_{k}}}\right)V_{i},\;Q_{i}=HW_{i}^{Q},\\ K_{i}=HW_{i}^{K},\;V_{i}=HW_{i}^{V}. \end{array}$$

In our implementation, we apply this with heads m=4 and $d_{k}=16$ while the total feature dimension is 64. Finally, we compress the output of the transformer into a latent representation $z\in R^{16}$ using a Dense Layer as follows:

(15) $$z=\phi (W_{2}\mathrm{MHA}\left(H\right)+b_{2}).$$

The decoder has a similar architecture as the encoder, that is, a hidden layer with 64 units and a linear output layer that reconstructs the input features:

(16) $$\hat{x}=W_{3}\phi \left(W_{4}z+b_{4}\right)+b_{3}.$$

The model parameters are optimized by minimizing the mean squared error of reconstruction. In this way, Equation (17) establishes the model parameters are learned with respect to minimizing the mean square error when the decoder reconstructs the data.

(17) $$L_{\mathrm{rec}}={∥x-\hat{x}∥}_{2}^{2},$$

Adam optimized and for 100 epochs with a batch size of 8. After training, the encoder alone is used to specify the latent representation learned z and projected in two dimensions by UMAP for visualization and exploratory analysis throughout the sessions.

(18) $$u=\mathrm{UMAP}\left(z\right)\in R^{2}.$$

The two-dimensional embeddings are then estimated not only throughout the session but also throughout the three therapy phases: (i) before-therapy, (ii) during-therapy, and (iii) after-therapy, along with the patient’s information, which allows us to qualitatively inspect the separation of the recordings, as well as the stability of the learned latent space.

### 2.9. Latent Space Metrics and Stability

This subsection presents the quality and stability criteria used to judge the learned latent representations in a test-agnostic way. Like the baseline VAE and transformer autoencoder, we first measure the reconstruction quality with the mean squared error (MSE) as follows:

(19) $$L_{\mathrm{rec}}=\frac{1}{N}\sum_{i=1}^{N}{∥x_{i}-{\hat{x}}_{i}∥}_{2}^{2},$$

which directly reflects to what extent the original EEG rhythm features are preserved by the encoder–decoder architecture. In addition to performance in reconstructing, the geometry of latent space is discussed by comparing with the compactness and dispersion. We quantify this using the variance of the embeddings,

(20) $$\mathrm{Var}\left(Z\right)=\frac{1}{N}\sum_{i=1}^{N}{∥z_{i}-\bar{z}∥}_{2}^{2},$$

and by intra- and inter-group distances calculated within therapy phases (pre-, during-, and post-therapy) between patients. If we have a grouping *G*, the variation within the group is established by the following equation:

(21) $$D_{\mathrm{intra}}\left(G\right)=\frac{1}{\left|G\right|}\sum_{i\in G}{∥z_{i}-{\bar{z}}_{G}∥}_{2}^{2},$$

where the distance from a group to its centroid is used to determine the separation between groups and is calculated by

(22) $$D_{\mathrm{inter}}\left(G_{a},G_{b}\right)={∥{\bar{z}}_{G_{a}}-{\bar{z}}_{G_{b}}∥}_{2}.$$

The latent space is stable if within the same phase or patient samples experience low $D_{\mathrm{intra}}$, while distinct phases or subjects have higher $D_{\mathrm{inter}}$.

To finally evaluate the extent to which local neighborhood relationships are retained after dimension reduction (for example, with UMAP), we calculate a k-nearest-neighbor (kNN) consistency score between some selected original and two-dimensional projected latent space:

(23) $$\mathrm{Cons}\left(k\right)=\frac{1}{N}\sum_{i=1}^{N}\frac{|N_{k}\left(z_{i}\right)\cap N_{k}\left(u_{i}\right)|}{k},$$

where $N_{k}\left(\cdot \right)$ means the k-th nearest neighbor set in the high-dimensional latent space z and, by analogy, in 2D embedding u. Together, these measures form a consistent methodological framework to verify the stability and separability of EEG representations before reporting experimental results.

## 3. Experimental Results

### 3.1. Experimental Setup

All of our experiments were conducted using Python 3 in Google Colab Pro, a flexible and reproducible cloud-based platform. Training and testing were carried out on an NVIDIA A100 GPU (Nvidia, Santa Clara, CA, USA), which ensured reliable optimization of deep models and the treatment of attention-based architectures. We also extended the system RAM, as a significant part of our processing pipeline involved handling multiple EEG sessions and high-dimensional latent representations, as well as multiple UMAP projections, without running out of memory. This configuration was used to provide consistent results across runs and rapid iteration during model development and analysis. Furthermore, all recordings were saved as text files compressed into a ZIP file. Every session was described by the average of the EEG rhythms: δ, θ, $\alpha_{↓}$, $\alpha^{↑}$, $\beta_{↓}$, $\beta^{↑}$, $\gamma_{↓}$ and $\gamma^{↑}$, as well as attention and meditation. The resulting matrix at the session level was normalized to z-score. The dataset consists of 94 EEG text files obtained from six patients (Patient 1–Patient 6) during three periods of this experiment: Before, During, and After. The number of recordings per patient is reasonably balanced, with 13 files for Patient 1 and 19 files for Patient 3, while the remaining patients contributed 14–17 files each. In total, there are 36 Before recordings, 29 During recordings, and 29 After recordings in the dataset, showing a slightly larger baseline (Before) segment. The recordings generated for each patient are divided among the three stages, thus allowing within-subject comparisons of the EEG signals over time according to the intervention protocol.

In order to ensure reproducibility, the dataset used in this research is ready to be downloaded through the following link: Google Drive Google Drive—EEG (https://drive.google.com/file/d/1WLPe6PFv67Twize7NcNNFMeKMaAOyLse/, accessed on 5 May 2026).

### 3.2. Evaluation of the Baseline Variational Autoencoder

As a first step, we trained a Baseline model with 16-unit hidden layer and 2D latent space, using MSE reconstruction loss and KL regularization term. The training applied Adam optimization, a batch of 8, and was performed for 200 epochs. During testing, the VAE was able to stabilize the reconstruction of the EEG feature vectors, which implied that a significant part of the rhythmic structure is preserved in the 2D latent representation. The latent space demonstrated some group level clustering of therapy phase, signifying that pre-, during-, and post-therapy sessions are to some extent able to be separated, although overlap remained, corresponding to the limited capacity of the model. These findings are consistent with the use of the VAE as a probabilistic baseline to evaluate the latent structure.

**Figure 9:**
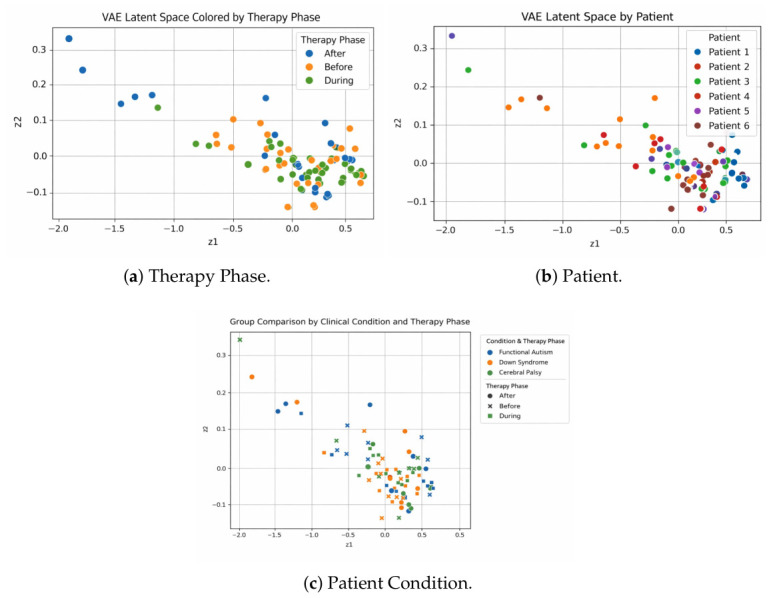
Two-dimensional VAE latent space visualizations under different grouping criteria.

For the VAE baseline, we obtained a 2D latent representation for each EEG session, denoted by $\left(z_{1},z_{2}\right)$ (Table 1). These coordinates constitute a compact description of the characteristics of EEG rhythms rather than clinical ones. These results suggest that the VAE learns a robust low-dimensional representation that enables direct comparison between patients and treatment phases (i.e., pre-, during, and post-therapy) and exploratory analysis of longitudinal evolution, as shown in Figure 9a. However, when it comes to cluster separation or clinical differences, numbers alone are not sufficient; rather, iterative visualization and a structured latent-space analysis are required. The 2D latent space colored by anonymized patient IDs is shown in Figure 9a,b, indicating substantial variability between subjects. Most sessions are located within a small central neighborhood, particularly for Patients 1 and 5, demonstrating a homogeneous structure of the EEG profiles. On the other hand, Patient 6 shows a more distinctive pattern in the dark region by occupying a more extreme region of the latent space, while Patient 2 shows moderate separation mainly along the negative axis $z_{1}$. The behavior of Patients 3 and 4 is intermediate, with an overlap in the central cluster. Taken together, these patterns suggest that the VAE potentially learns a clinically meaningful subject-specific structure that allows the identification of relevant latent profiles both homogeneous and atypical (i.e., without suggesting a clinical distinction). The latent space is plotted in Figure 9c according to clinical condition and therapy phase, illustrating the variability between conditions and the temporal stabilization of the distribution. Functional Autism has a loose pack (compared with Patient/Typical) before therapy; during and after the intervention, there is greater concentration towards the latent center. Down syndrome shows a complex pattern of behavior, with some stimuli eliciting considerably larger centralized effects than others. Cerebral palsy exhibits the tightest and most homogeneous latent structure throughout the therapy stages. Notably, the during-therapy phase produces the most uniform distributions even when comparisons are made over all conditions, asymptotically moving embeddings towards $\left(z_{1}\approx 0,z_{2}\approx 0\right)$, indicating a common stabilizing effect of therapy on EEG-derived latent spaces.

### 3.3. Transformer Autoencoder

**Figure 10:**
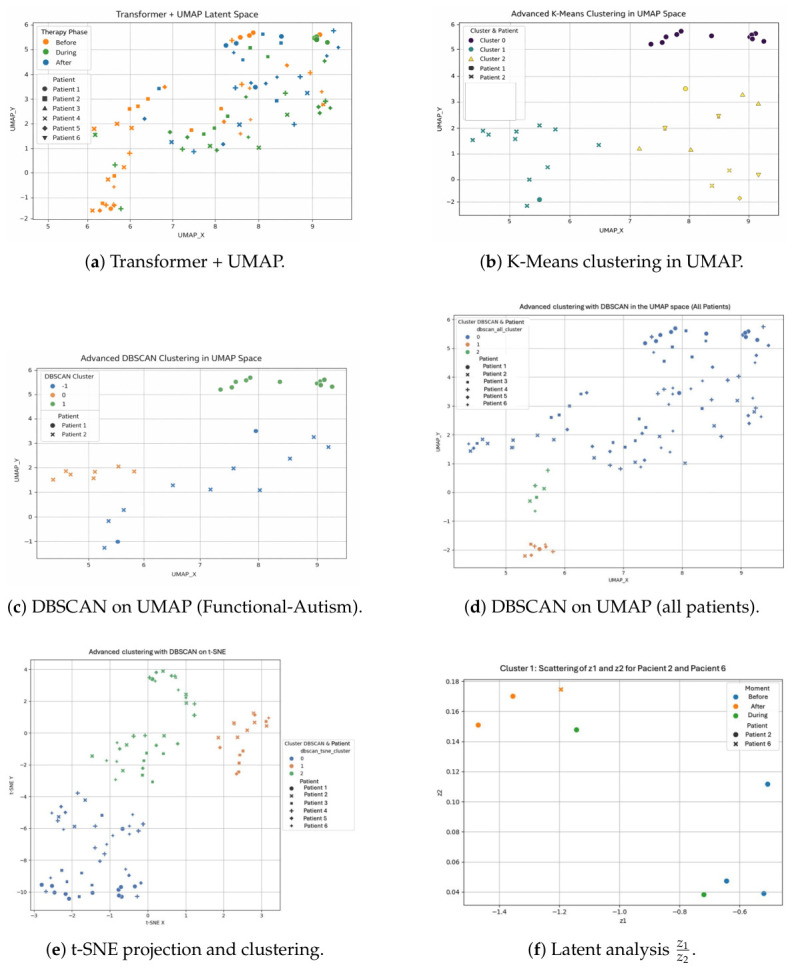
Latent-space visualizations under different projections and clustering strategies across therapy phases.

As a second step, or the Transformer autoencoder, the encoder used Dense (64, ReLU), then Multi-Head Attention (4 heads, key_dim = 16) followed by a 16D latent projection. The decoder is symmetric to the encoder with Dense (64, ReLU) and a linear output. Training was performed using Adam, batch size 8 and 100 epochs. UMAP was used to visualize latent embeddings such as $n_{neighbors}=15$, $\min_{dist}=0.1$, and $n_{components}=2$. The Transformer autoencoder resulted in more dense latent embeddings with a sharper phase-separation when visualized in the UMAP projection. More specifically, the 16D latent space provided denser within-phase clusters and greater distinctness between therapy phases due to superior performance in expressing cross-band relations. The Transformer autoencoder with a 16D latent space produced denser within-phase clusters and less overlap between therapy phases compared to the VAE baseline (2D latent space). We acknowledge that the comparison is confounded by the latent space dimensionality. Hence, we make no claim regarding architectural superiority and simply present the results as evidence that a higher-dimensional latent representation, regardless of architecture, enhances phase separation. The following experiments were performed to investigate how different nonlinear projections and clustering approaches manifest temporal structure, stability, and inter-individual variation in the latent space learned through therapy phases:

1. UMAP in latent space (Transformer + UMAP): The 16-dimensional embeddings learned by the Transformer autoencoder were reduced to two dimensions via UMAP for visualization of temporal trajectories. The resulting space demonstrates how Before sessions are spread throughout the entire $UMAP_{Y}$ range, generally lower on the scale (near 36), while During OCRs are more tightly bound together. Post sessions tend to move towards higher $UMAP_{X}$ and $UMAP_{Y}$ but with similar inter-individual variability; the same patients remain in clusters and some are still spread out, as shown in Figure 10a.
2. Clustering of K-Means clustering in UMAP: (k=3) Using the UMAP coordinates, a clear latent organization can be seen that aligns with the progression of therapy. One of the clusters has low $UMAP_{Y}$ values and high dispersion, mainly due to Before sessions. A second group, restricted to During and After sessions for relatively stable patients, sits at higher values of $\frac{UMAP_{X}}{UMAP_{Y}}$, and a third captures the transition between phases, Figure 10b.
3. DBSCAN on UMAP (Functional autism): The density based clustering with DBSCAN reveals both stable cores and outlier behavior in the UMAP space. A high-$UMAP_{Y}$ cluster indicates patients with stable post-therapy embeddings, while the rest of the points are distributed in mid-density and low-$UMAP_{Y}$ outliers, which means that some individuals exhibit lasting variability between sessions, Figure 10c.
4. DBSCAN on UMAP (all patients): When DBSCAN is performed on the entire cohort, there is a single dominant compact cluster that captures stable post-therapy representations shared among all patients. In contrast, smaller clusters with lower values in $UMAP_{Y}$ contain more sessions from subjects with greater variability, which are interpreted as transitional or atypical neurophysiological responses throughout the therapy phases, Figure 10d.
5. t-SNE projection and clustering: Similar results are supported using complementary t-SNE projections, which indicate that Before sessions are spread more evenly at higher *t*-SNE_*Y*_ values and converge towards During/After conditions. Applied to the t-SNE space, DBSCAN separates a tight post-therapy cluster from a more diffuse pre-therapy cluster, with an intercalated transitional cluster, Figure 10e.
6. Latent analysis $\frac{z_{1}}{z_{2}}$: Looking at the distribution of $z_{1}$ and $z_{2}$ in the most stable cluster, we observe that dispersion decreases significantly with therapy. Some patients show extremely negative $z_{1}$ values before therapy and a partial shift toward central latent values during and after the intervention, while others show more gradual turning points, Figure 10f.

## 4. Discussion

### 4.1. Interpretation of Results

**Figure 11:**
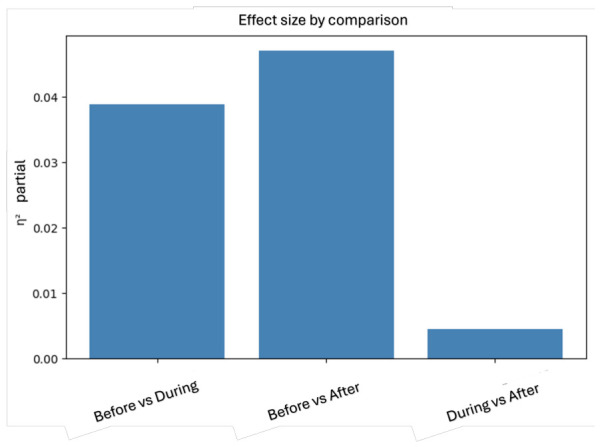
Partial effect size ($\eta_{\mathrm{partial}}^{2}$) for comparisons between Before, During and After conditions.

**Figure 12:**
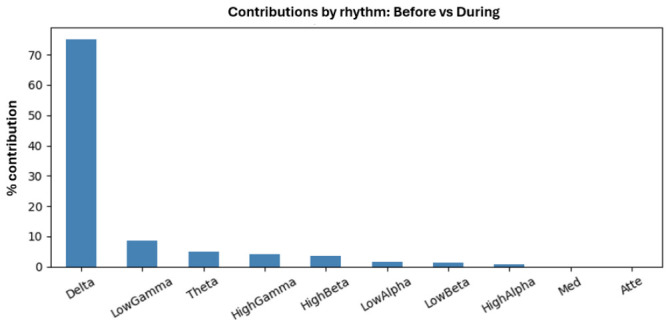
Contributions by rhythm: Before $v_{s}$ During.

A comprehensive analysis of multivariate EEG comparisons was performed between the Before, During, and After conditions, considering the magnitude of the effect using $\eta_{\mathrm{partial}}^{2}$, as well as its precision through 95% confidence intervals, and the relative contribution of the different EEG rhythms to each observed difference, as shown in Figure 11. For the Before vs. During comparison, a $\eta_{\mathrm{partial}}^{2}$ of 0.0388 was obtained, corresponding to a small to moderate effect, indicating that approximately 3.9% of the multivariate EEG variance was explained by the change between the two conditions. The 95% confidence interval was [0.0317, 0.0486], which does not include zero, confirming that the effect is statistically consistent, Figure 12. The analysis of contributions reveals a clear predominance of δ rhythms at 75.13%, followed by $\gamma_{↓}$ at 8.54%, θ at 4.94%, $\gamma^{↑}$ at 4.01%, and $\beta^{↑}$ at 3.61%. This suggests that the transition from Before to During therapy is primarily associated with changes in slow rhythms, especially δ. The Before vs. After comparison showed a larger effect size, with a $\eta_{\mathrm{partial}}^{2}$ of 0.0470, implying that approximately 4.7% of the total multivariate EEG variance changed between the two conditions, with a confidence interval of 95% [0.0384, 0.0635], indicating a robust effect. In this case, the contribution of the rhythms was more balanced, with θ standing out at 25.17%, $\gamma_{↓}$ at 24.72%, and $\beta^{↑}$ at 19.26%, followed by δ at 13.90% and $\gamma^{↑}$ at 8.88%. This suggests that there may be a broader reconfiguration of brain activity after the intervention. In contrast, the During vs. After comparison showed an $\eta_{\mathrm{partial}}^{2}$ = 0.0046, corresponding to a very small effect, with a 95% confidence interval [0.0032, 0.0086], indicating that both conditions exhibit highly similar EEG activity. Overall, these results show a change in EEG activity between the Before and After conditions, suggesting that brain activity tends to stabilize once therapy begins, with this pattern persisting in the subsequent phase. This finding opens up new opportunities to investigate EEG activity more deeply and broaden the scope of the proposed research.

On the other hand, we acknowledge that the main limitation of this proposal is the use of 10 aggregated EEG features input instead of raw temporal signals, which limits Transformer architecture substantially. Even so, the originality of this work does not come from simply running a transformer on any EEG data, but rather showing that, even in this low-dimensional feature-summary regime (as established by the designs of MLP bottlenecks in variational autoencoders), self-attention can reveal cross-band spectral interactions that a variance-exploration model cannot. While previous research mainly involves the use of VAEs for static EEG representations or raw signal reconstruction, we propose a comparison framework that is more clinically meaningful in a specific therapeutic context, dolphin-assisted therapy, where phase separation (pre/during/post), and latent stability are preferred over temporal fidelity. Therefore, although our method might be seen as a modest increment from an input-complexity point of view, it makes substantial progress in showing that Transformer-based autoencoders provide denser within-phase clusters and more interpretable prognostic trajectories, which we believe can generalize to other sensor-based condition-monitoring situations beyond the clinical domain.

The authors admit that a direct comparison to similar studies on neurological disorders is not given. This is because while there are public EEG datasets, none can be directly used nor reused due to the difference in dataset (dolphin-assisted therapy with single-channel EEG in Cerebral Palsy, Down Syndrome and Autism) and input features. Thus, the results are presented in terms of internal consistency and qualitative understandability rather than external validation. Current work should focus on the application of the proposed methodology to standard public EEG datasets (e.g., motor imagery, seizure detection) where cross-study comparison would be more appropriate.

### 4.2. Limitations

The present study has a number of limitations that should be considered. The generalizability of the findings beyond the six subjects in this dataset is limited, while the longitudinal depth of data across three years for 94 EEG sessions is a strength, no cross-validation scheme between subjects (e.g., leave-one-subject-out) was used, and the separation reported between therapy phases was primarily visual (UMAP/t-SNE projections) without statistical validation across multiple training runs. Thus, the clusters and trajectories reported in this study ought to be viewed as qualitative descriptors of neurophysiological state changes rather than clinically validated diagnostic or prognostic indicators. The interpretation of UMAP or t-SNE projections is known to be qualitative in nature. Quantitative metrics, including partial eta squared, reconstruction error, and intra/inter-group distances, were provided alongside additional clustering validation metrics that were not applied to the latent space embeddings. This limitation is acknowledged, and follow-up work will include quantitative statistical tests of this nature to better substantiate the observed clusters separations.

In relation to the lack of a healthy or sham-intervention control group, it has already been pointed out that Moreno Escobar et al. has taken into account the placebo effect in dolphin-assisted therapy. with an experimental design (2020) [21]. That study had a neurotypical normal control patient and important experimental conditions with or without the dolphin; the results showed that having the dolphin present was required for therapeutic efficacy. Hence, although the current study did not include a control group, the placebo effect has previously been accounted for in this treatment setting.

It is important to note that the baseline VAE and Transformer autoencoder (downsampled to create 2D latent maps) were designed with differing dimensions for their latent spaces (2D vs. 16D), in keeping with their respective architectural constraints: a low-dimensional bottleneck was necessary for the VAE to impose a Gaussian prior and prevent posterior collapse, while a high-dimensional latent space (16D) for the Transformer autoencoder allowed high capacity without regularization collapse due to self-attention encoder dynamics. As such, the comparison is more indicative of realistic design options for each architecture, rather than a contrived equalization of dimensions. The improvements in cluster separation and phase stability seen here are therefore attributed to the joint effect of the transformer architecture as well as its higher-dimensional latent capacity, allowing for inter-band spectral interactions that cannot be achieved with increased latent dimensions using a VAE.

The proposed framework leverages existing methods, such as variational autoencoders and transformer-based architectures, but addresses low-dimensional, clinically meaningful EEG representations. Unlike the majority of literature, based on raw temporal signals and emphasizing reconstruction fidelity, this works on aggregated spectral features on purpose in order to enhance interpretability and longitudinal stability over therapy stages.

This design decision is a methodological decision and not a limitation aligned with the clinical purpose of the study. For therapy monitoring situations, such as small and heterogeneous data sets, compact spectral representations provide robustness against noise and inter-session variability, which are common in EEG recordings. In this narrow feature space, a transformer-based autoencoder has proven to be advantageous by modeling cross-band interactions through self-attention and the identification of latent structures which are not captured by traditional MLP-based VAEs.

Consequently, the novelty of this work is not based on architectural invention alone but rather on showing that transformer-based representation learning still matters and makes sense even in simplified EEG feature regimes. We have highlighted this for bridging the gap between high-complexity deep learning models and practical and clinically interpretable applications around longitudinal therapy analysis.

## 5. Conclusions

### 5.1. General Conclusions

This study proposed a representation-learning framework to analyze EEG data throughout several therapy stages, employing a variational autoencoder, as well as a Transformer-based autoencoder. The evaluation results revealed that the Transformer-based model produces more structured and separable latent representations than the baseline VAE. These results suggest that self-attention mechanisms can capture the linkages among EEG spectral information accurately, facilitating enhanced differentiation between pre-, during-, and post-therapy phase. This results in a more interpretable depiction of neurophysiological changes from the intervention. Overall, the proposed method validates the potential contribution of latent representation learning as a means in a practical method of monitoring therapy progression and offers some numeric insights complementary to clinical evaluation.

### 5.2. Future Works

Future work includes extending the analysis to a larger number of subjects and different clinical profiles, including other cognitive deficits such as attention deficit disorder, to assess the generalizability of the patterns observed in the EEG. Furthermore, the incorporation of temporal and multiscale analyses will allow for a more detailed study of the evolution of brain activity during different phases of therapy, thus strengthening its application in clinical settings and neuroscientific analyses. In addition, a controlled ablation study could be involved with matched dimensionality to more precisely disentangle the individual contributions of architectural design (self-attention vs. MLP) and latent space dimensionality. Here, we present promising progress toward new EEG representation-learning approaches for therapy monitoring. In this way, future work on temporal sequence modeling will progress by departing from static band-averaged features. Methods to maintain intra-session temporal evolution and allow for genuine time-resolved neural-state monitoring (i.e., sliding windows, LSTMs, Transformer encoders with positional embeddings) will be investigated. Furthermore, the longitudinal dimension of this dataset will be used (94 sessions acquired over 3 years) to apply stringent subject-level validation. To assess generalization to unseen patients, a leave-one-subject-out scheme will be applied. For true diagnostic support, the authors will develop supervised classifiers trained on the learned latent representations for automated phase prediction (pre/during/post). For prognostic modeling, sessions per patient will be used to train a model that predicts post-therapy latent states from pre-therapy features in a regression or recurrent architecture conditioned on the temporal ordering of sessions. The authors would like to emphasize that this is an exploratory study and not meant to validate clinical diagnostic or prognostic tools. This framework is proposed with the intention of producing qualitative indicators for the monitoring of this therapy. Rigorous quantitative validation (such as subject level cross-validation, supervised classification) is still an important direction for future work.

## Figures and Tables

**Table 1:** 2D latent embeddings by patient and therapy moment.

Patient	Moment	Date	$z_{1}$	$z_{2}$
Patient 1	After	15 October 2024	0.325	−0.098
Patient 2	Before	17 October 2024	−0.520	0.039
Patient 3	After	17 October 2024	0.369	0.005
Patient 4	After	18 October 2024	0.354	−0.090
Patient 3	During	15 November 2022	0.057	−0.004

## Data Availability

The EEG corpus used in this study can be downloaded via the following link: https://drive.google.com/file/d/1WLPe6PFv67Twize7NcNNFMeKMaAOyLse (accessed on 31 March 2026). This corpus contains 94 EEG recordings stored as text files, collected from six patients (Patient1–Patient6) and distributed across three stages: 36 Before, 29 During, and 29 After recordings.
